# Healthcare Supply Chain Network Coordination Through Medical Insurance Strategies with Reference Price Effect

**DOI:** 10.3390/ijerph16183479

**Published:** 2019-09-18

**Authors:** Lingyu Gao, Xiaoli Wang

**Affiliations:** School of Economics and Management, Tongji University, Shanghai 201804, China; xiaoli-wang@tongji.edu.cn

**Keywords:** healthcare supply chain network, medical insurance reimbursement strategies, reference price effect, game theory, patient choice behavior

## Abstract

China has established the universal medical insurance system and individual out of pocket costs have decreased, however, the average healthcare expenditure of the Chinese population and the expenses of the whole society have increased substantially. One major challenge which impedes the progress of attaining sustainable development of the social healthcare system in China is that the number of hospital admissions is disproportionate. Superior hospitals are overcrowded, whereas subordinate hospitals are experiencing low admissions. In this paper, we apply the game theory model to coordinate the healthcare supply chain network, which is composed of the government, medical insurance fund, superior hospitals, subordinate hospitals and patients. Especially by taking the reference price effect into account, this paper analyzes different medical insurance reimbursement strategies and their influence on patient choice and the healthcare supply chain network. The result shows that the reference price effect increases the leverage of medical insurance, guides patients’ choice, optimizes the allocation of medical resources and reduces the medical expends. In comparison to a decentralized decision- making strategy, a centralized decision- making strategy can stimulate both superior hospital and subordinate hospital’s cooperative intentions which benefits the social healthcare system.

## 1. Introduction

China has successfully achieved universal health insurance coverage since 2011, representing the largest expansion of insurance coverage in human history [1]. The proportion of out of pocket cost for medical expense decreased from 34% in 2011 to 15% in 2018. However, patients’ average expenditure increased from 187.8 yuan to 268.3 yuan and the total medical expenses increased by 28.3% from 2011 to 2018 [2]. On the other hand, due to the rapid economic development in China, its citizens are becoming more health-conscious and there is a strong demand for high-quality medical services. In China, the healthcare supply chain network is composed of the government, medical insurance fund, medical institutions and patients. The medical institutions are classified as superior hospitals and subordinate hospitals [3]. Superior hospitals have advantages over subordinate hospitals in many aspects, such as access to advanced medical equipment and doctors’ professional ability. It is noteworthy that patients have the liberty to choose which hospital to seek treatment at. They tend to choose superior hospitals regardless of the type of disease they are suffering, resulting in the overcrowding of superior hospitals. Consequently, subordinate hospitals are experiencing low admission rates [4,5]. In fact, for patients with common diseases, it is unnecessary for them to seek treatment in superior hospitals as they would be able to obtain the proper treatment in subordinate hospitals. Furthermore, because superior hospitals charge more than subordinate hospitals for the same medical treatment [6], the total medical expense is increased. All these circumstances reflect the serious social problems of the efficiency and fairness in the whole healthcare system [7].

The most important factor is that the distribution of medical resources is disproportionate and the cooperation among healthcare supply chain network participants has not yet been established. Consequently, due to the lack of standardization, patients are faced with a dilemma which impedes their ability to make informed decisions. Thus, a mandate the need to resolve this issue by making medical treatment accessible, lowering the cost of medical services and implementing a reasonable allocations of medical resources.

Medical insurance is an effective tool to optimize the allocation of resources and coordinate healthcare system, and by setting different reimbursement strategies it also can guide patients’ choice [8]. Medical insurance can reduce the patients’ expenses through the help of reimbursements, which ease the medical burden of patients and solve the problem of the high cost of medical services [9]. When considering a medical insurance reimbursement strategy, there are two basic rules. First, different reimbursement standards for different diseases are set according to medical policy [10]. Second, reimbursements are also different depending on different medical institutions [11,12]. The government regulates the use of reimbursements and the overall profits of the healthcare supply chain network. Meanwhile, the total insurance expenses are fully considered when formulating insurance strategies. In this way, the optimal medical benefits can be obtained with the minimum insurance expenditures [5], so different medical insurance policies should be set according to the medical treatment situation of different hospitals, which not only benefit different hospitals, but also restrict patients’ choice, which achieves the purpose of controlling medical expenses.

Under the guidance of the new medical reform policy, medical insurance is no longer just a reimbursement, but also a powerful tool to coordinate the whole healthcare supply chain network. The medical insurance reimbursement can promote the vertical cooperation between the two medical institutions, optimize the allocation of resources, realize resource sharing and complementary advantages, effectively reduce the cost of medical services and improve patients’ utility [13,14,15].

Therefore, the implementation of the medical insurance reimbursement strategy to improve rational utilization of medical resource and coordinate healthcare supply chain network is a research direction that is worthy of our attention. Although medical insurance reimbursement strategies have been extensively studied during the past several decades, reference price effect, an important marketing phenomenon, has not been considered in the healthcare supply chain network [16]. The reference price is the price expectation of the consumer for a product, which is derived from the customer’s purchase experience, price information of the product or other non-price factors [17]. The direct effect of reference price is to generate the consumers’ perceived price. Consumers usually have a specific reference price when making purchase decisions [18]. The influence of reference price on consumers’ decision-making is reflected in the following aspects: when consumers want to buy a commodity, if the market price of the commodity is lower than the psychological reference price, their inner sense of ‘profit’ will prompt them to buy; on the contrary, if the market price is higher than the psychological reference price, they will refuse to buy due to the ‘sense of loss’ [19]. In medical systems the reference price effect also exists [20]. The medical reference price system has been put into practice in most countries and proved to be an effective way for reimbursement standard management [21]. For instance, Germany implemented a health care reform act in 1989 and took the lead in implementing integrated pricing and compensation in its legal health insurance system—a reference price system [22]. The reference price system indirectly controls the medical services price by adjusting the level of medical insurance reimbursements and increasing the sensitivity of patients to the price. Moreover, the reference price effect improves the elasticity of demand and the competition in the supplier market, resulting in the reduction of medical service cost. The reference price provides a reference standard and basis between the patients, hospitals and the government or insurance company [23]. The reference price effect, as a factor to stimulate the selection of patients, plays a fundamental role in promoting the coordination of the medical service market.

For patients, the reference price can influence their demand and decision-making process which ultimately guides their choice behaviour. China’s medical insurance system clearly stipulates the starting standard, the maximum payment limit and the reimbursement ratio. Under the reference price effect, patients tend to choose hospitals with higher reimbursement rates, which adjusts the reasonable allocation of medical insurance funds to some extent [24]. This would restrict the behaviour of patients, balance the interests of all parties, and coordinate the health service supply chain network. For this reason, it can influence patients’ choice, and able to solve the problems of overcrowded hospitals and enhance admission rates at subordinate hospitals. In this study, common diseases that can be treated equally well in both subordinate and superior hospitals are discussed. Patients have right to choose an appropriate hospital, but an important factor is that if patients want to get the normal medical insurance reimbursement ratio at a superior hospital, they must be refered by the doctor of the subordinate hospital and obtain the transfer certification [25]. By doing so, patients can enjoy a high ratio of the reimbursement of about 85%. Contrary, patients without the transfer certification can only enjoy a reimbursement ratio of 45% [26]. Due the variation in reimbursement ratio stipulated by different hospitals, the patients’ reference price effect will affect their choice of hospitals. Meanwhile, medical insurance can play a vital role in promoting the implementation of patient triage and hierarchical diagnosis and treatment.

In this paper, a dynamic game theory model is applied to coordinate healthcare supply chain network. Especially, reference price effect has been integrated with the model to study the influence it has on patients’ choice of hospitals.

This study also investigated different medical insurance reimbursement strategies and proposed a cooperation mechanism to coordinate the healthcare supply chain network, which guides the patients to make reasonable choice, optimizes the allocation of network resources, reduces medical expense, and benefits the social healthcare system.

The reminder of the paper is organized as follows: Section 2 presents the literature review; Section 3 introduces the problem statement and basic model framework; Section 4 deduces the model and analyze the relationship between the main variables. Section 5 analyzes the impact of medical insurance reimbursement on healthcare supply chain network coordination. Section 6 numerical simulation. Section 7 presents the conclusions and future research directions.

## 2. Literature Review

The literature related to this paper focuses mainly on healthcare supply chain network, medical insurance and reference price effect. This study was approved by Health and Planning Commission of Qixian (No. QWZ201803005) on 5 March 2018.

### 2.1. Healthcare Supply Chain Network

Healthcare supply chain networks are a popular topic, and many researchers have been interested in investigating this area in recent years. The healthcare supply chain network is provided by a variety of product and service enterprises, including medical consumables, pharmaceuticals, catering, waste management, information technology, vehicle fleet management, general supplies [13] and the main participants include the government, hospitals, doctors and patients [8]. Due to the critical role supply chains play in the healthcare industry, different methods have been suggested in the literature for cost reduction [14]. What is more, studies on the healthcare area of operation management and supply chain management are still relevant. Most of the studies in both fields still consider the supply chain operation and technology in normal circumstances [16]. 

The research on healthcare supply chain network usually adopts game theory to build a theoretical model and analyze the behavior and benefit of each participants [27]. Sinha et al. [28] built a game model of medical quality and medical costs and they proposed a medical insurance strategy that could encourage medical service providers to reduce costs and avoid extra compensation. Gardner et al. [29] used game theory to analyze the main body of a new type of rural cooperative medical insurance and found that arousing the enthusiasm of patients’ participation and strengthening the supervision of patients in action are keys to the success of the new medical policy. Landry et al. [30] studied incentive theory to explore the feasibility of medical insurance incentives and believed that medical insurance reimbursement should take into full consideration the patients’ psychological acceptability to improve the visiting rate of subordinate hospitals as well as the accessibility and availability of medical services. In summary, it can be inferred from these studies that introducing game theory as a strategic tool can produce positive effects at organizational of healthcare supply chain network [31]. A review of domestic and foreign literatures on the coordination of vertical supply chain shows that most of them establish relevant mathematical models from a theoretical perspective and use game theory methods for analysis [32]. 

In terms of coordinating the healthcare supply chain network, medical insurance reimbursement is a basic tool. Different strategies are discussed to promote vertical cooperation, optimize allocation of resource, realize resource sharing, complementary advantages, effectively reduce the cost of medical services and improve service quality [16,33,34]. Lillrank et al. [35] considered that hospitals usually have different price control strategies and used the model to analyze patients’ selection of quality care. Zhong et al. [36] indicated that medical insurance goals should be positioned to common disease and frequently-occurring disease, so as to improve the basic rural medical and healthcare system accessibility and availability, especially to reduce the cost of treatment. Jayaram et al. [37] found that when the marginal cost of medical services is high, the existence of medical insurance can increase the utility of patients. 

At present, compared with the horizontal competitions in medical institution of the healthcare supply chain network, vertical cooperation has attracted extensive attention. Zhang et al. [11] studied the impact of a service cost sharing strategy of upstream and downstream enterprises on the profit of the entire supply chain by establishing the vertical cooperation model between enterprises. Lehoux et al. [38] successively studied the effects of vertical integration and vertical structure on the cooperation between upstream and downstream enterprises and the interests of the whole supply chain. Cao, Wan et al. [39] studied vertical cooperation strategies in a green supply chain and analyzed the reimbursement effect of the same government expenditure under different strategies. Jaber et al. [40] discussed the competition and cooperation theories of each member of the supply chain under different consumer strategies. Samuel et al. [14] pointed out the problems caused by vertical integration of differentiated products by investigating the structure of bilateral monopoly market. On the basis of considering the financial reimbursement policy, Panda et al. [41] systematically compared and discussed the game model of cooperation between manufacturers and suppliers in three types of supply chains. In addition, most current game theory research models are based on static cooperation [42] and in order to achieve more scientific supply chain coordination, it is very important to study the vertical supply chain model of dynamic cooperation [43].

### 2.2. Medical Insurance 

Universal health insurance coverage was successfully achieved by China in 2011 which marks the largest expansion of insurance coverage in human history. It is rare that universal health insurance coverage can be found in developing countries, nevertheless the largest developing country—China with 1.3 billion population has achieved this [4]. China’s achievement is impressive, but it is still facing daunting challenges. The following gives a brief introduction of China’s medical insurance policy to help better understand the research background of this paper. The priority for medical insurance is to mitigate financial risks faced by patients. According to China’s Minister of Health, even with health insurance coverage, the financial risk remained high [44]. To rectify the issue, the government has increased the amount of public insurance reimbursement with the objective of reducing out-of-pocket payment to 30% of total health expenditures by 2018 [45]. The primary objective of medical insurance is to improve the medical service quality. The government has increased its investment in healthcare system, but the effect is still unknown. Due to the rising income among the Chinese people, there is a strong demand for higher quality healthcare. To meet the demand, the medical service quality should be improved [46]. The medical insurance is used as a tool to coordinate the hospitals reimbursement to improve the revenue so as to improve the medical quality [47].

In China there are three basic public medical insurance systems: New Rural Cooperative Medical Scheme (NRCMS), Urban Resident Basic Medical Insurance (UBMI) and Urban Employee Basic Medical Insurance (UEBMI) [48]. In January 2016, the state council issued opinions on integrating the NRCMS and UBMI into the Urban and Rural residents’ unified Basic Medical Insurance system (URBMI) [49]. The next step the government will establish and improve the connection and integration the medical insurance systems into one of the Citizen Basic Medical Insurance (CBMI) [50]. Because of the frequent identity changes, besides the health department, civil affairs and other departments will establish a coordination mechanism, establish and improve the cohesion and integration of various systems. 

Furthermore, the government has improved the rate of reimbursement for the urban and rural residents’ medical insurance. On 13 May 2019, the state health insurance administration and the ministry of finance issued a notice on ensuring basic medical care for urban and rural residents, calling for a steady increase [25]. The reimbursement rate for URBMI improved from 50% to 60% and the hope exists to establish the same level of reimbursement rates for all the citizens in the future [24,51].

### 2.3. Reference Price

Researchers focus on healthcare system of supply chain and the medical insurance, however the basic participant, the patient, who produces the demand of the whole system is not discussed. Patients’ choice is the most important factor for the coordination of healthcare supply chain networks. The reference price effect, as an important factor influencing patients’ choice, is not taken into consideration [52]. Reference price has been researched since at least the 1980s and it has been a mature research theory in the field of psychology and marketing [53]. Bambauer [54] indicated that the reference price framework was consistent with several psychological theories of consumer behavior and price perception, including the adaptation-level theory and the assimilation contrast theory. Reference price is crucial for consumers’ choice, product pricing and coordination of the entire supply chain [20]. Dye et al. [17] believed that consumers usually have specific reference prices when making purchase decisions. A common conceptualization views reference price as a predictive price expectation that is shaped by consumers’ prior experience and current purchase environment. As argued by Choi [55], reference price represents a consumer’s product evaluation and it is influenced by various factors such as price (including the historical price, the suggested retail price, the rival product’s price, etc.), quantity, advertisement. According to Mazumdar et al. [52] the influence of a reference price on consumer demand can be due to the dynamic comparison between reference price and current market price. Xu et al. [56] further suggested that an actual price either higher or lower than the reference price has a different impact on consumers’ product evaluation. 

Extensive studies have examined the influence of reference price effect on consumer purchase and enterprise operation strategy and how it can be measured or modeled [52,53]. The concept of reference point has also been extended to other stimuli such as price promotions and product quality [57]. Some researchers have also developed models of consumer choice by using the economic theory and game theory [18,58]. Others have considered reference price effects in modeling competitive behavior of firms and have developed managerial guidelines for retailers and manufacturers [59]. Despite the importance of reference price concept, no comprehensive framework exists has systematically examined reference price effect in healthcare supply chain network. 

In the medical industry, the reference price effect will directly affect the patient’s decision of seeking medical treatment, and it is the key factor for patients’ choice of hospital [54,60]. Therefore, the introduction of reference price effects into the innovation model of vertical coordination of healthcare supply chain network can help participants make more scientific and reasonable decisions.

## 3. Problem Description and Model Building

### 3.1. Problem Description 

**Figure 1:**
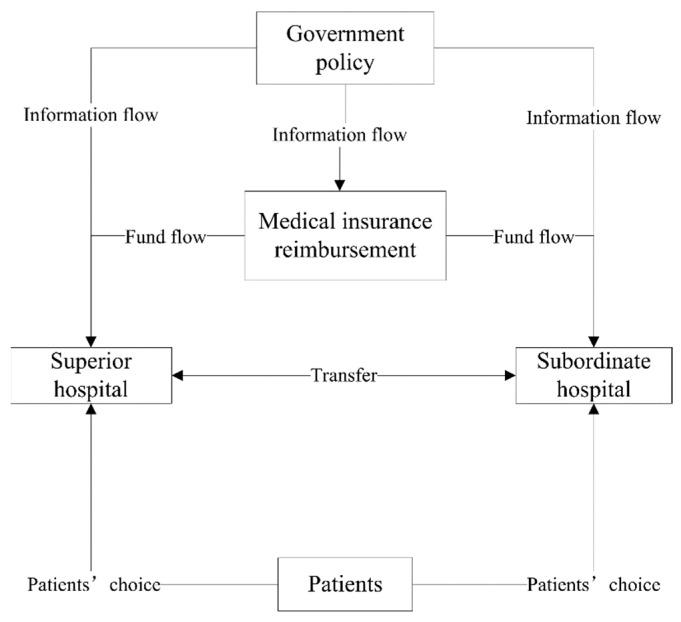
Healthcare supply chain network.

This section develops a healthcare supply chain network consisting of the government, medical insurance fund, medical service institutions (including superior hospitals and subordinate hospitals) and patients. The network is shown in Figure 1. The government carries out overall management of whole healthcare system and formulates medical insurance reimbursement policies. The public goods nature of medical services determines the necessity for the government to participate in the basic medical service provision. As the manager of the medical security system, the government provides policy support and financial support for the medical security system, and effectively supervises the medical market [61]. In 2009, the new medical reform plan clarified the leading role of the government in basic medical services [62]. As clients of medical services and supervisors of medical activities, medical insurance institutions are an important link in medical service transactions and are a lever to promote the reform of the entire medical and health system.

Patients are the consumers of medical services, and they drive the medical service supply chain. Thus, patients’ utility satisfaction is an important index for the performance evaluation of the health service supply chain [63]. Providing satisfactory services to patients is the goal of all nodes in the health service supply chain. The health service supply chain starts from the needs of patients, promoting the flow of logistics, information flow and capital flow, and ultimately enable patients to receive high-quality, safe and effective medical and health services [64]. However, due to the information asymmetry between doctors and patients in the medical service market, the doctors and hospitals with more professional information are in a dominant position, while patients are in a passive position, they can only rely on health care providers to determine their purchase of medical services [65]. Patients have no active bargaining rights, but they can determine which hospital to choose. When patients decide which medical institutions to visit, both medical service quality and the price are the two major considerations that they will take into account [15]. However, as patients have subjective knowledge of the differences between in two-tiers medical institutions, they are more likely assumed that the superior hospital has better curative effect, even though both superior and subordinate hospitals are capable at treating the same disease. In this regard, the patients’ acceptable reference price will be much higher than subordinate hospital [66]. 

In the new medical reform policy, the government guides patients to choose reasonable medical institutions by regulating medical insurance reimbursement strategy and hopes to maximize the interests of all participates. So the government requires superior hospitals to provide certain support subsidies through medical insurance to the subordinate hospital to promote patients diversion and make full use of medical resources [67]. Finally, depend on the different medical insurance reimbursement, medical service prices and the prior judgment on the curative effect of different medical institutions, patients form a psychological reference price and choose the hospital to maximize their own benefits.

### 3.2. Benchmark Model Building 

According to Zhang [68] consumers adjust their reference price according to their own memory effect and psychological cognition. The reference price function is given as follows $\left(\dot{r}\left(t\right)=\frac{dr\left(t\right)}{dt}\right)$:

(1) $$\dot{r}\left(t\right)=\gamma \left(p\left(t\right)-r\left(t\right)\right),\;r\left(0\right)=r_{0}$$

where *r*(*t*) is the reference price, *p*(*t*) is the current price which means the average medical service price (including medicine fee, diagnosis fee, etc.) required for a patient to complete the treatment course, t∈[0, +∞) is the specific time when patient going to the hospital. *γ* denotes the impact of patients’ memory effect. 0≤γ≤1, a higher *γ* implies the consumer has short term memory and less satisfaction with this choice. $r\left(0\right)=r_{0}$ is a constant, which is usually affected by patients’ satisfaction, medical service quality and price similar.

The government set the current medical insurance reimbursement strategy for superior hospital is *S_h_*(*t*), and for subordinate hospital is *S_b_*(*t*). In the new medical reform policy, the patient’s psychological reference price will slightly increase because of the reimbursement, therefore, patients’ reference price function is re-defined as:

(2) $$\dot{r}\left(t\right)=\gamma \left(p\left(t\right)-r\left(t\right)\right)+\theta_{b}S_{b}\left(t\right)+\theta_{h}S_{h}\left(t\right),\;\;r\left(0\right)=r_{0}$$

where $\theta_{b},S_{b}$ represent the effect of subordinate and superior hospitals’ reimbursement on the reference price, respectively. Generally, investments in medical insurance reimbursement can enhance a hospital’s image, which further increases the consumer’s valuations on its service quality [53]. Thus $\theta_{h}>0$ and $\theta_{b}>0$ are assumed. Based on this, the patient consultation demand function in the healthcare supply chain network with the reference price effect is obtained:

(3) $$D\left(t\right)=\alpha -\beta p\left(t\right)+\delta \left[r\left(t\right)-p\left(t\right)+\theta_{h}S_{h}\left(t\right)+\theta_{b}S_{b}\left(t\right)\right]$$

D(t)=α−βp(t) represents the patients’ demand in the general medical service market without reference price effect. α>0 denotes the potential market capacity, β>0 donates the price elasticity of demand. δ>0 represents the impact of reference price on demand. A higher δ means patients are more sensitive to the gap between the reference price and the actual medical service price [55]. In special cases, δ=0 means the reference price effect is not taken into consideration. 

The cost functions contained in the medical insurance for superior hospital and subordinate hospital are respectively set as $C_{h}=\frac{1}{2}\eta_{h}S_{h}{\left(t\right)}^{2}$ and $C_{b}=\frac{1}{2}\eta_{b}S_{b}{\left(t\right)}^{2}$ ($\eta_{h}\geq 1$ and $\eta_{b}\geq 1$ are the reverse measurement coefficients of respective efficiency in superior hospital and subordinate hospital) [69,70].

In the healthcare supply chain network with the reference price effect, the psychological price of patients is affected by the memory effect when they make choices, that is, when they choose medical service institutions, they not only depend on their current state, but also depend on the past experience [55]. The utility function of patient is:

(4) $$U=U\left(x\right)+\dot{r}\left(t\right)+F\left(t\right)$$

Patients have quasi-linear preference [17], the function (4) can be rewritten as:

(5) $$U=\ln\left(x\right)+\dot{r}\left(t\right)+F\left(t\right)$$

where *x* represents patient’s assets, x=w−p+θs(t), *w* represents the initial asset of the patient and obey the uniform distribution on [0, *W*]. *F*(*t*) represents the patient’s health status at the moment, F∈[0, 1], 0 represents the patient is in sick, 1 represents the patient is healthy. $F^{0}\left(t\right)=0$ means the patient does not go to see the doctor. 

At the same time, besides patients’ choice, the government guides the superior and subordinate hospitals to sign the agreement on the secondary distribution. The superior hospital provides certain financial subsidies and technical support to the subordinate hospital, in order to effectively alleviate the situation of its medical resources shortage and improve the overall healthcare benefits. It is assumed that the average marginal profits of superior and subordinate hospitals are $\rho_{h}$ and $\rho_{b}$. φ∈[0, 1) is set as the specific proportion coefficient of secondary distribution, then the profit function of superior and subordinate hospital are:

(6) $$\pi_{h}=\int_{0}^{\infty }e^{-\phi t}\left(\rho_{h}D_{h}\left(t\right)-\frac{1}{2}\eta_{h}S_{h}^{2}\left(t\right)-\frac{1}{2}\varphi \eta_{b}S_{b}^{2}\left(t\right)\right)dt$$

(7) $$\pi_{b}=\int_{0}^{\infty }e^{-\phi t}\left(\rho_{b}D_{b}\left(t\right)-\frac{1}{2}\left(1-\varphi \right)\eta_{b}S_{b}^{2}\left(t\right)\right)dt$$

where ϕ>0 represent the corresponding discount rate. Then the total profit function of the healthcare supply chain network is:

(8) $$\pi_{sc}=\int_{0}^{\infty }e^{-\phi t}\left(\left(\rho_{h}D_{h}+\rho_{b}D_{b}\right)-\frac{1}{2}\eta_{h}S_{h}^{2}\left(t\right)-\frac{1}{2}\eta_{b}S_{b}^{2}\left(t\right)\right)dt$$

Now we build the dynamic game model and use the Hamiltonian function to optimize the whole supply chain system. Based on Equations (6) and (7), the Hamiltonian function of the superior and subordinate hospital in the healthcare supply chain network are as follows:

(9) $$H_{h}=\rho_{h}\left(\alpha -\beta p+\delta \left(r-p\right)+\theta_{h}S_{h}+\theta_{b}S_{b}\right)-\frac{1}{2}\eta_{h}S_{h}^{2}-\frac{1}{2}\varphi \eta_{h}S_{b}^{2}+\nu_{h}\left(\gamma \left(p-r\right)\right)+\theta_{h}S_{h}+\theta_{b}S_{b}$$

(10) $$H_{h}=\rho_{h}\left(\alpha -\beta p+\delta \left(r-p\right)+\theta_{h}S_{h}+\theta_{b}S_{b}\right)-\frac{1}{2}\left(1-\varphi \right)\eta_{h}S_{b}^{2}+\nu_{h}\left(\gamma \left(p-r\right)\right)+\theta_{h}S_{h}+\theta_{b}S_{b}$$

where $v_{h}$ and $v_{b}$ are the respective adjoint variables, indicating the impact of the reference price on the revenue of superior and subordinate hospitals. Relevant notations used in this paper are explained in Table 1.

## 4. Model Analysis

### 4.1. Decentralized Decision Model 

In the decentralized decision model, government makes the medical insurance reimbursement policy depending on hospitals optimal decision to maximize their respective interests [71].

#### 4.1.1. Nash Equilibrium Strategy (NS)

In this strategy, superior and subordinate hospitals do not know each other’s decision. Both participates conduct simultaneous action and obtain their respective optimal decisions to maximize their own interests, which is eligible with Nash equilibrium game conditions. In the process, the medical insurance reimbursement for the superior hospital is $S_{h}^{ns}$, and for the subordinate hospital is $S_{b}^{ns}\left(t\right)$.

Using the optimal control theory of dynamic programming, we can obtain the solution as follows:

*Proposition 1*. The optimal Nash equilibrium strategy of medical insurance reimbursement for the superior hospital is:

(11) $$S_{h}^{ns}\left(t\right)=\frac{1}{n_{h}}\left(\theta_{h}\rho_{h}+\frac{\theta_{h}\delta \rho_{h}}{\phi +\gamma }\right)$$

In the same way, medical insurance reimbursement for the subordinate hospital is:

(12) $$S_{b}^{ns}\left(t\right)=\frac{1}{n_{b}}\left(\theta_{b}\rho_{b}+\frac{\theta_{b}\delta \rho_{b}}{\phi +\gamma }\right)$$

*Proof*: Take the first partial derivative of $S_{h}$ in Equation (9) and set it equal to zero:

(13) $$\frac{\partial H_{h}}{\partial S_{h}}=0\overset{}{\Leftrightarrow }\eta_{h}S_{h}=\theta_{h}\rho_{h}+\theta_{h}\nu_{h}$$

where:

(14) $$\dot{\nu }=\phi \nu_{h}-\frac{\partial H_{h}}{\partial r}=\left(\phi +r\right)\nu_{h}-\delta \rho_{h}$$

Solving Equation (14), we get the following general solution:

(15) $$\nu_{h}=Ae^{\left(\phi +\gamma \right)t}+\frac{\delta \rho_{h}}{\phi +\gamma }$$

*A* is the parameter to be determined, if A≠0, $v_{h}$ goes to infinity as time goes on, which does not match the reality, so A=0. In the NS strategy, there is no cooperation, so φ=0.

Substituting Equation (15) into Equation (13), we get:

(16) $$S_{h}^{ns}\left(t\right)=\frac{1}{\eta_{h}}\left(\theta_{h}\rho_{h}+\frac{\theta_{h}\delta \rho_{h}}{\phi +\gamma }\right)$$

Similarly, considering Equation (10), the solution is:

(17) $$S_{b}^{ns}\left(t\right)=\frac{1}{\eta_{b}}\left(\theta_{b}\rho_{b}+\frac{\theta_{b}\delta \rho_{b}}{\phi +\gamma }\right)$$

According to the analysis of *Proposition 1*, the following conclusions can be obtained:

It can be seen from Table 2 and Table 3 that $S_{b}^{ns}$ is positively correlated with $\theta_{b}$ and $S_{h}^{ns}$ is positively correlated with $\theta_{h}$. At the same time, $S_{h}^{ns},\;S_{b}^{ns}$ are both positively correlated with *δ*. This means that the medical insurance reimbursement will increase with the increasing of reference price effect, and the cognition of overpricing will be alleviated, which solves the problem of the high cost of getting medical treatment.

Since $S_{h}^{ns}$ is positively correlated with $\rho_{h}$ and $S_{b}^{ns}$ is positively correlated with $\rho_{b}$, we get that medical insurance reimbursements can improve the marginal benefit. $S_{h}^{ns}$ has a negative correlation with $\eta_{h}$ which means it is positively correlated with the service efficiency of hospitals, as is $S_{b}^{ns}$ with $\eta_{b}$. This conclusion indicates that medical insurance reimbursements can improve the service efficiency of hospitals and reduce patients’ waiting time.

#### 4.1.2. Stackelberg Strategy Analysis (SS)

We consider that the superior hospital is in a dominant position for it is a high level medical institution and it has the priority in decision making of medical insurance reimbursement strategy, which fits the Stackelberg game theory conditions. In the process, the medical insurance reimbursement for the superior hospital is $S_{h}^{ss}\left(t\right)$, and for the subordinate hospital it is $S_{b}^{ss}\left(t\right)$. The reimbursement rate for a subordinate hospital is φ.

According to Equations (9) and (10), using the optimal control theory of dynamic programming, we get the equilibrium solution of $S_{h}^{ss}\left(t\right)$ and $S_{b}^{ss}\left(t\right)$ in Stackelberg game, which is shown in the following Proposition:

*Proposition 2*: In Stackelberg game theory, the optimal medical insurance reimbursement of superior hospital is:

(18) $$S_{h}^{ss}\left(t\right)=\frac{1}{\eta_{h}}\left(\theta_{h}\rho_{h}+\frac{\theta_{h}\delta \rho_{h}}{\phi +\gamma }\right)$$

Similarly, the optimal medical insurance reimbursement of a subordinate hospital is:

(19) $$S_{b}^{ss}\left(t\right)=\frac{1}{\eta_{b}\left(1-\varphi \right)}\left(\theta_{b}\rho_{b}+\frac{\theta_{b}\delta \rho_{b}}{\phi +\gamma }\right)$$

The proof of *Proposition 2* is similar to that of *Proposition 1*.

According to the Stackelberg equilibrium solution in *Proposition 2*, the optimal medical reimbursement rate for the subordinate hospital can be found, which is shown in *Proposition 3*.

*Proposition 3***:** In Stackelberg game theory, the optimal reimbursement rate for a subordinate hospital is:

(20) $$\varphi =\{\begin{array}{cc} \frac{2\rho_{h}-\rho_{b}}{2\rho_{h}}, & 2\rho_{h}>\rho_{b}\\ 0 & others \end{array}$$

*Proof*: Substituting Equations (18) and (19) into Equation (2), we can get:

(21) $$\dot{r}\left(t\right)=\frac{dr\left(t\right)}{dt}=\gamma \left(p-r\right)+\theta_{h}S_{h}^{ss}+\theta_{b}S_{b}^{ss}$$

Solving Equation (21) we obtain its general solution:

(22) $$r\left(t\right)=C_{0}e^{-\gamma t}+r_{sss}$$

where $r_{sss}=p+\left(\theta_{h}S_{h}^{ss}+\theta_{b}S_{b}^{ss}\right)/\gamma$

Setting t=0 and combining this with Equation (22), we get:

(23) $$r_{0}=C_{0}+r_{sss}\overset{}{\Leftrightarrow }r_{0}-r_{sss}$$

Then, Equation (22) is substituted into the profit function of the superior hospital (6) to obtain:

(24) $$\pi_{h}=\rho_{h}\left(\frac{\alpha -\beta p+\delta \left(r_{sss}-p\right)+\theta_{h}S_{h}^{ss}+\theta_{b}S_{b}^{ss}}{\phi }+\frac{\delta C_{0}}{\phi +\gamma }\right)-\frac{\eta_{h}S_{h}^{ss}-\varphi \eta_{b}S_{b}^{ss}}{2\phi }$$

Substituting Equations (18) and (19) into Equation (24), and taking its first partial derivative and then setting it equal to zero:

(25) $$\frac{d\pi_{h}}{\varphi }=0\underset{}{\Leftrightarrow }\varphi =\frac{2\rho_{h}-\rho_{b}}{2\rho_{h}}$$

Finally, we combine the domain of φ∈[0,1) and we get optimal reimbursement rate for a subordinate hospital.

*Proposition 3* shows that in Stackelberg game theory, the superior hospital’s medical insurance reimbursement $S_{h}^{ss}\left(t\right)$ remains unchanged, while the reimbursement rate for the subordinate hospital φ is mainly based on the marginal benefit of both participants $\rho_{h},\;\rho_{b}$.

### 4.2. Centralized Decision Model(CS)

In a centralized decision model, the government establishes the medical insurance reimbursement policy to maximize the overall interest of the whole healthcare supply chain network [72]. Under the current supply chain structure of vertical integration of medical consortia, if a binding cooperation agreement is reached on the medical policy project, the coordination between the superior and subordinate hospitals in the healthcare supply chain network will be carried out [41]. Then, we use a centralized decision model and according to Equation (8), its Hamiltonian function is:

(26) $$H_{sc}=\left(\rho_{h}+\rho_{b}\right)\left(\alpha -\beta p+\delta \left(r-p\right)+\theta_{h}S_{h}+\theta_{b}S_{b}\right)-\frac{1}{2}\eta_{h}S_{h}^{2}-\frac{1}{2}\eta_{b}S_{b}^{2}+\nu_{sc}\left(\gamma \left(p-r\right)+\theta_{h}S_{h}+\theta_{b}S_{b}\right)$$

where $v_{sc}$ is an adjoint variable that denotes the reference price effect impact on the supply chain. According to the Equation (26), using the optimal control theory and dynamic programming, we get the best medical insurance subsidies for superior and subordinate hospital, which is shown as Proposition 4.

*Proposition 4*: In the centralized decision mode, the medical insurance reimbursement of the superior hospital is:

(27) $$S_{h}^{cs}\left(t\right)=\frac{1}{\eta_{h}}\left(\theta_{h}\left(\rho_{h}+\rho_{b}\right)\right)+\frac{\theta_{h}\delta \left(\rho_{h}+\rho_{b}\right)}{\phi +\gamma }$$

Similarly, the medical insurance reimbursement of subordinate hospital is:

(28) $$S_{b}^{cs}\left(t\right)=\frac{1}{\eta_{b}}\left(\theta_{b}\left(\rho_{h}+\rho_{b}\right)\right)+\frac{\theta_{b}\delta \left(\rho_{h}+\rho_{b}\right)}{\phi +\gamma }$$

The proof of *Proposition 4* is similar to that of *Proposition 1*.

According to the above results, when taking the reference price effect into account in the centralized decision model of the supply chain, the reimbursement amount of medical insurance for superior and subordinate hospitals depends on the patients’ reference price and the marginal profit value of medical institutions. Different from the decentralized decision model, in the vertical integration of medical systems, each medical institution would consider the marginal benefits of its related upstream and downstream institutions when making decisions, which improves the coordination of the healthcare supply chain network.

Therefore, *Proposition 4* indicates that, in coordination of the supply chain, the medical treatment insurance reimbursement is not only determined by the reference price effect, but also the all cooperative medical institutions’ marginal profit. In this way we can promote the integration of medical institutions and maximize the benefit of entire healthcare supply chain network.

## 5. Healthcare Supply Chain Network Coordination

In this section, we will discuss how the reference price effect influence the healthcare supply chain network coordination on patients’ choice and the utility of healthcare system. 

### 5.1. Patient Choice in Healthcare Supply Chain Network

Patients make medical treatment choices based on their own utility. According to Equation (5):

The utility of patients to not to see the doctor is:

$$U_{0}=ln\left(x\right)+\dot{r}\left(t\right)+F_{0}\left(t\right)$$

The utility of patients to choose the subordinate hospital is:

$$U_{b}=ln\left(x\right)+\dot{r}\left(t\right)+F_{b}\left(t\right)$$

The utility of patients who choose the superior hospital is:

$$U_{h}=ln\left(x\right)+\dot{r}\left(t\right)+F_{h}\left(t\right)$$

The analysis of the patients’ behavior is as follows:

1) The situation that patients do not to see the doctor.

Obviously, $F_{b}\left(t\right)$ and $F_{h}\left(t\right)$ are greater than $F_{0}\left(t\right)$, so $U_{0}$ is less than $U_{b}$ and $U_{h}$ which means when people fell sick, they will choose to see the doctor.

2) The situation that patients choose the subordinate hospital.

In this situation, the utility function of patients should satisfy $U_{b}>U_{h}$, that is:

(29) $$\ln\left[w-p_{b}+\theta_{b}s_{b}\left(t\right)\right]+\dot{r}\left(t\right)+F_{b}\left(t\right)>ln\left[w-p_{h}+\theta_{h}S_{h}\left(t\right)\right]+\dot{r}\left(t\right)+F_{h}\left(t\right)$$

(30) $$\overset{}{\Rightarrow }\ln\left[\frac{w-p_{b}+\theta_{b}S_{b}\left(t\right)}{w-p_{h}+\theta_{h}S_{h}\left(t\right)}\right]>F_{h}\left(t\right)-F_{b}\left(t\right)$$

Because patients have a priori subjectivity, they believe that the superior hospital has better service quality than a subordinate hospital for the same medical treatment, so set $F_{h}\left(t\right)-F_{b}\left(t\right)=a$ and 0<a<1, then we get that if:

$$w<\frac{e^{a}\left[p_{h}-\theta_{h}S_{h}\left(t\right)\right]-p_{b}+\theta_{b}S_{b}\left(t\right)}{e^{a}-1}$$

the patient will choose the subordinate hospital and the number is:

(31) $$n_{b}=D\left(t\right)P\left(w\leq \frac{e^{a}\left[p_{h}-\theta_{h}S_{h}\left(t\right)\right]-p_{b}+\theta_{b}S_{b}\left(t\right)}{e^{a}-1}\right)\;=D\left(t\right)\frac{e^{a}\left[p_{h}-\theta_{h}S_{h}\left(t\right)\right]-p_{b}+\theta_{b}S_{b}\left(t\right)}{W\left(e^{a}-1\right)}$$

3) The situation that patients choose the superior hospital.

In this situation, the utility function of patients should satisfy $U_{h}>U_{b}$ that is:

(32) $$\ln\left[w-p_{h}+\theta_{h}s_{h}\left(t\right)\right]+\dot{r}\left(t\right)+F_{h}\left(t\right)>ln\left[w-p_{b}+\theta_{b}S_{b}\left(t\right)\right]+\dot{r}\left(t\right)+F_{b}\left(t\right)$$

(33) $$\overset{}{\Rightarrow }\ln\left[\frac{w-p_{b}+\theta_{b}S_{b}\left(t\right)}{w-p_{h}+\theta_{h}S_{h}\left(t\right)}\right]<F_{h}\left(t\right)-F_{b}\left(t\right)$$

The number of patients that choose the superior hospital is:

(34) $$n_{b}=D\left(t\right)P\left(w>\frac{e^{a}\left[p_{h}-\theta_{h}S_{h}\left(t\right)\right]-p_{b}+\theta_{b}S_{b}\left(t\right)}{e^{a}-1}\right)\;n_{b}=D\left(t\right)P\left(w>\frac{e^{a}\left[p_{h}-\theta_{h}S_{h}\left(t\right)\right]-p_{b}+\theta_{b}S_{b}\left(t\right)}{e^{a}-1}\right)$$

From Equation (31) we know that investing in $S_{b}$ led to the $n_{b}$ increasing and investing in $S_{h}$ led to the $n_{b}$ decreasing, which illustrates that increasing the subordinate hospital’s medical insurance reimbursement will improve its number of patient visits but increasing the reimbursement for the superior hospital will decrease the subordinate hospital’s number of patient visits. The same conclusion can be obtained from Equation (34), so medical insurance reimbursement is an important factor that influences patients’ choice. 

Next we go to a further discussion of how the δ affects the $n_{b},n_{h}$.

*Corollary 1***.** The number of patients in subordinate hospital will increase with a higher the reference price effect of patients. 

*Proof*: From Equation (31) we know that:

$$n_{b}=D\left(t\right)P\left(\frac{e^{a}\left[p_{h}-\theta_{h}S_{h}\left(t\right)\right]-p_{b}+\theta_{b}S_{b}\left(t\right)}{W\left(e^{a}-1\right)}\right)$$

Let:

$$Z=\frac{e^{a}\left[p_{h}-\theta_{h}S_{h}\left(t\right)\right]-p_{b}+\theta_{b}S_{b}\left(t\right)}{W\left(e^{a}-1\right)}$$

$n_{b}=D\left(t\right)Z$ take the first derivative of $n_{b}$ with respect to δ.

(35) $$\frac{\partial n_{b}}{\partial \delta }=\frac{\partial D\left(t\right)}{\partial \delta }Z+D\left(t\right)\frac{\partial Z}{\partial \delta }$$

According to Equation (3):

(36) $$\frac{\partial D}{\partial \delta }=r-p+\theta_{b}\frac{\partial S_{b}}{\partial \delta }+\theta_{h}\frac{\partial S_{h}}{\partial \delta }$$

Take the first derivative of *z* with respect to δ:

(37) $$\frac{\partial Z}{\partial \delta }=\left(-e^{a}\frac{\partial S_{h}}{\partial \delta }+\frac{\partial S_{b}}{\partial \delta }\right)/W\left(e^{a}-1\right)$$

Substitutinge (36) and (37) into (35) we get:

(38) $$\begin{array}{c} \frac{\partial n_{b}}{\partial \delta }=\left(r-p+\theta_{b}\frac{\partial S_{b}}{\partial \delta }+\theta_{h}\frac{\partial S_{h}}{\partial \delta }\right)\frac{e^{a}\left[p_{h}-\theta_{h}S_{h}\left(t\right)-p_{b}+\theta_{b}S_{b}\left(t\right)\right]}{W\left(e^{a}-1\right)}\\ \;\;\;\;+(\alpha -\beta p\left(t\right)+\delta \left(r\left(t\right)-p\left(t\right)+\theta_{h}S_{h}\left(t\right)\right)\\ \;\;\;\;+\theta_{b}S_{b}\left(t\right))\left[\left(-e^{a}\frac{\theta_{h}\partial S_{h}}{\partial \delta }+\frac{\theta_{b}\partial S_{b}}{\partial \delta }\right)/W\left(e^{a}-1\right)\right] \end{array}$$

To determine the optimal value, the second derivative is obtained:

(39) $$\frac{\partial^{2}n_{b}}{\partial \delta^{2}}=\frac{2\left[r\left(r-p\right)\eta_{h}\eta_{b}+\theta_{h}^{2}\rho_{h}\eta_{b}+\theta_{b}^{2}\rho_{b}\eta_{h}\right]\left(\eta_{b}\theta_{b}\rho_{b}-e^{a}\eta_{h}\theta_{h}\rho_{h}\right)}{W\left(e^{a}-1\right)\eta_{h}^{2}\eta_{b}^{2}\gamma^{2}}$$

It can be seen from the assumption in the third part that the service price, reference price effect, cost and marginal benefit of the superior hospitals are higher than that of the subordinate hospitals, that is, $\eta_{b}\theta_{b}\rho_{b}<\eta_{h}\theta_{h}\rho_{h},$ as 0<a<1 we can get that $e^{a}>1$, so $\eta_{b}\theta_{b}\rho_{b}-e^{a}\eta_{h}\theta_{h}\rho_{h}<0$. As a convex function, the maximum value can be obtained at $\frac{\partial n_{b}}{\partial \delta }=0$

According to the proposition in the fourth part, we solve Equation (38) in three different game theory strategies. The optimal δ corresponding maximum value of $n_{b}$ under three different strategies can then be obtained, respectively:

(40) $$\delta_{0}^{ns}=\frac{\eta_{h}\eta_{b}\left(e^{a}p_{h}+p_{b}\right)+\eta_{h}\theta_{h}\rho_{h}+e^{a}\eta_{b}\theta_{b}\rho_{b}\gamma }{\lambda_{h}\rho_{h}+\lambda_{b}\rho_{b}}$$

(41) $$\delta_{0}^{ss}=\frac{\eta_{h}\eta_{b}\left(e^{a}p_{h}+p_{b}\right)+\left(1-\varphi \right)\eta_{h}\theta_{h}\rho_{h}+e^{a}\eta_{b}\theta_{b}\rho_{b}\left(\gamma +\varphi \right)}{\lambda_{h}\rho_{h}+\lambda_{b}\rho_{b}}$$

(42) $$\delta_{0}^{sc}=\frac{\eta_{h}\eta_{b}\left(e^{a}p_{h}+p_{b}\right)+\left(1-\varphi \right)\eta_{h}\theta_{h}\left(\rho_{h}+\rho_{b}\right)+e^{a}\eta_{b}\theta_{b}\left(\rho_{h}+\rho_{b}\right)\left(\gamma +\varphi \right)}{\lambda_{h}\lambda_{b}\left(\rho_{h}+\rho_{b}\right)}$$

Since,0<δ<1, $p_{h}>\rho_{h},p_{b}>\rho_{b}$,$\;\eta_{h}\geq 1,$ $\eta_{b}\geq 1$ and γ,φ,θ are all between 0 and 1, we get $\delta_{0}>1$.It can be seen that the value of $\eta_{b}$ is increased with the increase of δ in the interval of [0,1] and the maximum value is obtained At δ=1.

Therefore, with the increase of patients’ reference price effect, the utility of patients choosing a subordinate hospital for treatment is improved, so that more patients receive treatment in subordinate hospitals. Similarly, the number of patients visiting the superior hospital decreases which relieves its pressure. Thus, the reference price effect increases the leverage of medical insurance reimbursement and effectively promotes patients’ utility. Furthermore, the reference price effect can guide patients to choose appropriate medical institutions to relieve the pressure of superior hospital, and at the same time coordinate the healthcare supply chain network.

### 5.2. The Utility of Healthcare System

The profit of the healthcare supply chain network system under different strategies can be obtained according to Equations (6)–(8), but for the whole healthcare system, we not only expect the highest profit of the supply chain. As a social service institution, the healthcare system provides a guarantee for people’s life and health, rather than being a profit-making institution. The real purpose is the optimal overall benefit of the society.

Since this supply chain is only for a certain common disease, if D(t)>α means that the resource provided for this disease is more than needed, which results in a surplus of medical resources. Based on the current situation of the lack of medical resources in China, when medical resources are idle, it means that there has more resources to serve for other types of diseases, which is usually a supplement to the medical resources for difficult and complicated diseases, thus increasing the benefits.

At this time, when calculating the profit of supply chain, the idle resources of the hospital are counted into the total revenue and the total utility benefit function of the healthcare supply chain network system is as follows:

(43) $$W=\{\begin{array}{cc} \tau \pi_{sc}+U & D<a\\ \tau \left(\pi_{sc}+\left(D-n\right)p\right)+U & D>a \end{array}$$

where τ is the conversion coefficient of profit to the supply chain utility [33]. τ>0 it means that the higher the supply chain profit the higher medical service utility we have.

## 6. Numerical Analysis

The data from a region of China is used to analyze effect of reference price on medical insurance reimbursement strategy and how it influences the patients’ choice and utility of healthcare supply chain network. We use three different game theory methods, that is, decentralized decision making methods of the Nash equilibrium and Stackelberg, and centralized decision making method.

According to the situation in the survey area, the specific values of the model parameters are set as $r_{h}=135,\;\;r_{b}=90,\;\;p_{h}=150,\;\;p_{b}=100,\;\;\gamma =0.3,\;\;W=1350,\;\;a=0.5,\;\;\theta_{h}=0.6,\;\;\theta_{b}=0.5,\;\;\alpha =300,\;\beta =1,\eta_{h}=1.25,\;\;\eta_{b}=1,\;\;\phi =0.3,\;\;\tau =0.8,\;\;\rho_{h}=128,\;\;\rho_{b}=80.$

Then the subsidy rate of subordinate hospital φ=0.2 can be calculated according to Equation (27).

**Figure 2:**
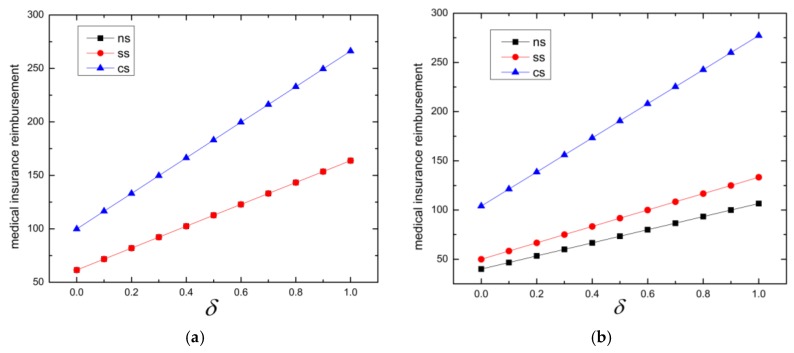
Medical insurance reimbursement under different reference price effects: (**a**) reimbursement for superior hospital; (**b**) reimbursement for subordinate hospital.

First, we calculate the medical insurance reimbursement for superior hospital and subordinate hospital under different reference price effects which is shown in Table 4 and we can see the variation tendency in Figure 2.

It can be seen from Figure 2 that medical insurance reimbursements increase as the reference price effect increases. The patients’ psychological needs and reference price effect become the main factors that affect the medical decision making. What is more, in Table 4 we can clearly conclude that centralized decision making can get more medical insurance reimbursements than decentralized decision making for both subordinate hospitals and superior hospitals.

**Figure 3:**
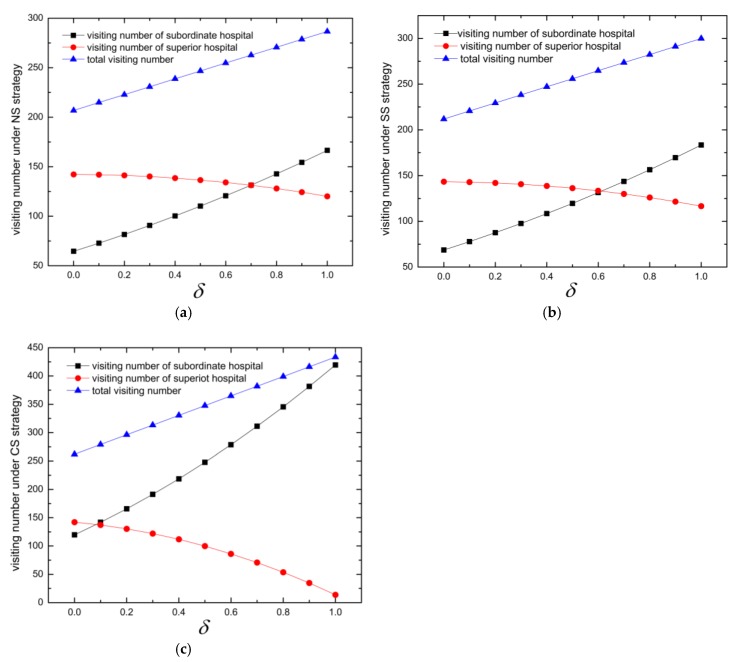
Number of visitors with reference price effects under different strategies: (**a**) Nash strategy (NS); (**b**) Stackelberg strategy (SS); (**c**) Centralized strategy (CS).

Secondly, we calculate the patient visiting number of superior and subordinate hospitals under different reference price effects, which is shown in Table 5 and the variation tendency can be seen in Figure 3. As seen in Figure 3, it can be intuitive get that the total number of visiting patients rise with the increase of δ. At the same time, the number of patients of the superior hospital decreases and that of the subordinate hospital increases, which illustrates that affected by the reference price effect and medical insurance strategy, patients tend to be more reasonable, which improves the number of patients visiting the subordinate hospital and reduces the pressure on the superior hospital under all the medical insurance strategies and the centralized strategy performs best in this area, as seen from Table 5. Furthermore, medical resources are used more reasonably used and this lets more patients have access to the healthcare system and get their treatment at the acceptable price.

**Figure 4:**
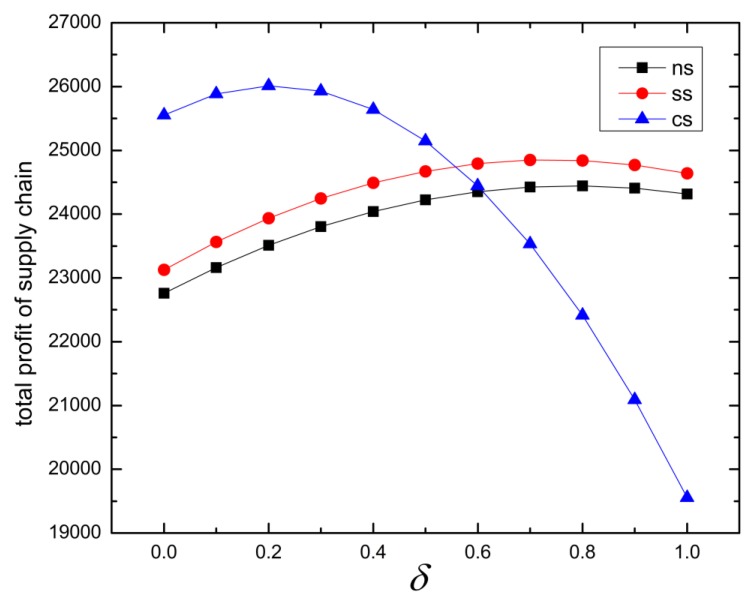
Total profit of the supply chain under different strategies.

Finally, we calculate the total profit of supply chain under different strategies, which is shown in Table 6 and the tendency can be seen in Figure 4.

When D(t)<α, medical resources are in short supply, the total income increases with the increase of the reference price effect in all strategies. Therefore, medical insurance investments not only increase patients’ willingness to see a doctor, but also increase the profits of medical institutions, and achieves a win-win situation. When D(t)>α, it means medical resources are idle. Under the CS strategy, the total profit for this certain disease is reduced while the total medical cost will decrease, which solves the problem that the medical insurance investment continues to increase but the total medical cost of patients increases. In terms of the overall medical system, common diseases can be basically cured in subordinate hospitals, which is a rational use of resources. The medical resources of superior hospitals can be used to treat relatively complex diseases, and the overall utility of the medical system will improve. The total utility under the different decision making of the healthcare supply chain network is shown in Table 7.

**Figure 5:**
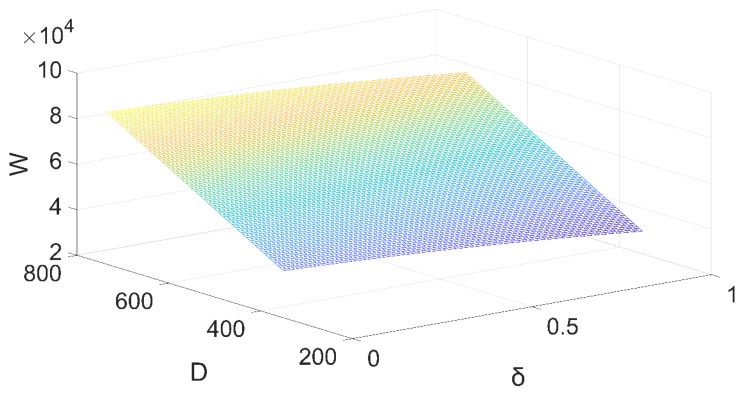
Total utility of healthcare supply chain network under the centralized decision.

The total number of patients and the total social benefits of the whole medical system are significantly greater than with decentralized decisions. In other words, through the coordination of the vertical integration of healthcare supply chain network can maximize the benefits of entire healthcare system δ. The variation tendency of total utility of healthcare supply chain network under the centralized decision can be seen in Figure 5.

## 7. Conclusions and Future Research

### 7.1. Conclusions

In this paper, we establish a dynamic game theory model with reference price effect and analyze the influence of medical insurance reimbursement strategies on healthcare supply chain network coordination. The results show that:

1) The medical insurance reimbursement strategy has a positive correlation with patients’ reference price effect. A higher reference price effect can get more reimbursement and other factors such as the marginal profit and service efficiency also influence reimbursement. The government should take full consideration of these factors and use the insurance reimbursement as a useful tool to push hospitals to improve service quality, reduce cost, and control the growth of health expenditures.
2) Taking advantage of the reference price effect in medical insurance can guide the patients’ choice. Patients make rational choices depending on different medical insurance reimbursement strategies. Hospitals get different reimbursements and use this to improve their service quality and optimize their medical resources. Under the new medical reform policy, the government improves the medical insurance reimbursements which can improve the patients’ reference price effect. With the increase of patients’ reference price effect, the utility of patients choosing subordinate hospitals for treatment improves, so that more patients receive treatment in subordinate hospitals. Similarly, the number of patient visits to superior hospitals decreases, which can help to rectify the problem of overcrowding. In this way the medical insurance can guide patients’ choice. These all alleviate the pressure of patient overcrowding in superior hospitals and boost the number of patients visiting subordinate hospitals. Furthermore, taking advantage of reference price effects can improve the effective use of medical resources and give more patients access to the healthcare system for treatment at an acceptable price.
3) The reference price effect can improve the utility of healthcare supply chain network under all the different medical insurance strategies. Furthermore, compared with the decentralized decision making of the Nash equilibrium and Stackelberg game, centralized decision making can strengthen the cooperation intention among different medical institutions, reduce the total medical expenses and improve the utility of the whole healthcare supply chain network. As to what can be seen from the numerical analysis: 1) In Table 4 under the centralized decision section, both the subordinate and superior hospital can get more reimbursement under centralized decisions than decentralized decisions of NS and SS. The additional reimbursement will increase the willingness of cooperation between superior and subordinate hospitals. 2) In Table 5, under the centralized decision section, it illustrates that when the number of patient admissions to a subordinate hospital increases, the admission rate decreases at the superior hospital. Under all the medical insurance strategies the centralized strategy performs best in this area. Furthermore, the average price of subordinate hospitals is lower than at superior hospitals, but the reimbursement is higher, which will reduce the patients’ expenses for medical services. 3) It also can be seen from Table 7 that the total utility under the centralized decision making of the healthcare supply chain network is higher than the decentralized one. In conclusion, the centralized decision scenario can further help facilitate a more efficient way of allocating medical resources. With a more strategic allocation of medical resources, healthcare services will be more accessible for patients at affordable prices, so vertical integration of the healthcare supply chain network with centralized decision provides the best outcome for the healthcare supply chain network.

### 7.2. Implications and Limitations

In order to ensure the sustainable development of healthcare supply chain network, the government should take full consideration of the reference price effect and exert its leverage when making its medical insurance reimbursement strategies, in order to make the medical insurance fund investment obtain the biggest economic returns. The medical insurance reimbursement can be used to optimize the allocation of medical resources. Subordinate hospitals should get more reimbursement to improve their service quality so patients are more likely to choose the subordinate hospitals, which reduces medical expense, and improves patients’ satisfaction. In healthcare supply chain network management, the government should play a leading role and use centralized decision making to balance the interests of all participants. All in all, by implementing a vertical integration supply chain under centralized-decision, we can realize the rational utilization of medical resources, improve the medical service quality, reduce medical expenditures and develop a sustainable healthcare supply chain network.

One limitation of this paper is that the research considers a common disease and does not include serious or life-threatening diseases or an emergency. Furthermore, the research data is from one geographical area which may have its own particular economic conditions. Therefore, we could expand the scope of the research by conducting a comparative study of a serious disease, and also to test the model in different situations.

### 7.3. Future Research Suggestions

In the future research, it would perhaps be interesting to study the following aspects: (i) The factors affecting the patients’ psychological reference price can be further developed. For example, patients’ characteristics and their medical knowledge. (ii) Refine the medical insurance and adopt different medical insurance reimbursement proportions for different medical services (including surgical treatment, nursing inspection and equipment) and different diseases to improve the utility of the medical insurance.

## Figures and Tables

**Table 1:** Notations.

Parameters	Description
*r*(*t*)	reference price of patients
*p*(*t*)	unit service price
*t*	specific time when patient going to the hospital
*γ*	impact of patients’ memory effect
*S*(*t*)	medical insurance reimbursement
*x*	patient’s asset
*w*	initial assets of the patient
*θ*	medical insurance reimbursement effect on the reference price
*D*(*t*)	patients consultation demand
*α*	base market size
*β*	price elasticity of demand
*δ*	impact of reference price on demand
*ρ*	average Marginal revenue
*C*	cost in medical insurance
*η*	reverse measurement coefficients of respective efficiency
*U*	utility of patient
*F*	patient’s health status
*φ*	specific proportion coefficient of secondary distribution
*π*	profit
*ϕ*	corresponding discount rate
*n*	actual number of patients’ admission
*v*	accompanying variables

Superscripts *ns*, *ss*, *cs* mean the medical insurance strategy under the Nash, Stackelberg and centralized model of game theory respectively. Subscripts *b*, *h* represent the superior hospital and subordinate hospital respectively.

**Table 2:** Relationship between superior hospital insurance reimbursement and parameters.

	Parameters	$\theta_{h}$↑	δ↑	$\rho_{h}$↑	$\eta_{h}$↑
Reimbursement					
$S_{h}^{ns}$		↑	↑	↑	↓

**Table 3:** Relationship between subordinate hospital insurance reimbursement and parameters.

	Parameters	$\theta_{b}$↑	δ↑	$\rho_{b}$↑	$\eta_{b}$↑
Reimbursement					
$S_{b}^{ns}$		↑	↑	↑	↓

**Table 4:** Medical insurance reimbursement for superior and subordinate hospital under different reference price effects.

δ	$S_{h}^{ns}$	$S_{b}^{ns}$	$S_{h}^{ss}$	$S_{b}^{ss}$	$S_{h}^{cs}$	$S_{b}^{cs}$
0.00	61.44	40.00	61.44	50.00	99.84	104.00
0.10	71.68	46.67	71.68	58.33	116.48	121.33
0.20	81.92	53.33	81.92	66.67	133.12	138.67
0.30	92.16	60.00	92.16	75.00	149.76	156.00
0.40	102.40	66.67	102.40	83.33	166.40	173.33
0.50	112.64	73.33	112.64	91.67	183.04	190.67
0.60	122.88	80.00	122.88	100.00	199.68	208.00
0.70	133.12	86.67	133.12	108.33	216.32	225.33
0.80	143.36	93.33	143.36	116.67	232.96	242.67
0.90	153.60	100.00	153.60	125.00	249.60	260.00
1.00	163.84	106.67	163.84	133.33	266.24	277.33

**Table 5:** Patients visiting number of superior and subordinate hospitals under different reference price effects.

δ	$n_{b}^{ns}$	$n_{h}^{ns}$	$D^{ns}$	$n_{b}^{ss}$	$n_{h}^{ss}$	$D^{ss}$	$n_{b}^{cs}$	$n_{h}^{cs}$	$D^{cs}$
0.00	64	142	206	68	143	211	119	142	261
0.10	72	141	214	77	142	220	141	137	279
0.20	81	141	222	87	141	229	165	130	296
0.30	90	140	230	97	140	238	191	122	313
0.40	100	138	238	108	138	247	218	111	330
0.50	110	136	246	119	136	255	247	99	347
0.60	120	134	254	131	133	264	278	86	364
0.70	131	131	262	143	129	273	311	70	381
0.80	142	127	270	156	125	282	345	53	399
0.90	154	124	278	169	121	291	381	34	416
1.00	166	120	286	183	116	299	419	13	433

**Table 6:** Total profit of the supply chain under different strategies.

δ	$\pi_{ns}$	$\pi_{ss}$	$\pi_{cs}$
0.00	22,760.82	23,128.96	25,552.43
0.10	23,163.00	23,563.60	25,885.91
0.20	23,510.40	23,935.21	26,012.04
0.30	23,803.02	24,243.78	25,930.83
0.40	24,040.86	24,489.32	25,642.28
0.50	24,223.92	24,671.83	25,146.39
0.60	24,352.21	24,791.31	24,443.16
0.70	24,425.72	24,847.75	23,532.59
0.80	24,444.44	24,841.16	22,414.68
0.90	24,408.39	24,771.54	21,089.42
1.00	24,317.57	24,638.88	19,556.83

**Table 7:** Total utility of the supply chain under different strategies.

δ	$W_{ns}$	$W_{ss}$	$W_{cs}$
0.00	32,902.73	33,221.73	36,808.27
0.10	33,461.44	33,817.85	37,706.09
0.20	33,985.93	34,375.26	38,480.61
0.30	34,476.20	34,893.95	39,131.82
0.40	34,932.27	35,373.93	39,659.73
0.50	35,354.11	35,815.20	40,064.34
0.60	35,741.75	36,217.75	40,345.65
0.70	36,095.17	36,581.58	40,503.66
0.80	36,414.37	36,906.70	40,538.36
0.90	36,699.36	37,193.11	40,579.76
1.00	36,950.14	37,440.80	40,618.86
